# Simulation and Modeling of Novel Electronic Device Architectures with NESS (Nano-Electronic Simulation Software): A Modular Nano TCAD Simulation Framework

**DOI:** 10.3390/mi12060680

**Published:** 2021-06-10

**Authors:** Cristina Medina-Bailon, Tapas Dutta, Ali Rezaei, Daniel Nagy, Fikru Adamu-Lema, Vihar P. Georgiev, Asen Asenov

**Affiliations:** 1 Device Modelling Group, School of Engineering, University of Glasgow, Glasgow G12 8LT, UK; Tapas.Dutta@glasgow.ac.uk (T.D.); Ali.Rezaei@glasgow.ac.uk (A.R.); Daniel.Nagy@glasgow.ac.uk (D.N.); Fikru.Adamu-Lema@glasgow.ac.uk (F.A.-L.); Asen.Asenov@glasgow.ac.uk (A.A.); 2 Nanoelectronics Research Group, Departamento de Electrónica y Tecnología de Computadores, Universidad de Granada, 18071 Granada, Spain

**Keywords:** integrated simulation environment, drift-diffusion, quantum correction, Kubo-Greenwood, non-equilibrium Green’s function, nanowire transistors (NWT), tunnel FETs (TFET), Negative Capacitance FETs (NCFET), resonant tunneling diodes (RTD)

## Abstract

The modeling of nano-electronic devices is a cost-effective approach for optimizing the semiconductor device performance and for guiding the fabrication technology. In this paper, we present the capabilities of the new flexible multi-scale nano TCAD simulation software called Nano-Electronic Simulation Software (NESS). NESS is designed to study the charge transport in contemporary and novel ultra-scaled semiconductor devices. In order to simulate the charge transport in such ultra-scaled devices with complex architectures and design, we have developed numerous simulation modules based on various simulation approaches. Currently, NESS contains a drift-diffusion, Kubo–Greenwood, and non-equilibrium Green’s function (NEGF) modules. All modules are numerical solvers which are implemented in the C++ programming language, and all of them are linked and solved self-consistently with the Poisson equation. Here, we have deployed some of those modules to showcase the capabilities of NESS to simulate advanced nano-scale semiconductor devices. The devices simulated in this paper are chosen to represent the current state-of-the-art and future technologies where quantum mechanical effects play an important role. Our examples include ultra-scaled nanowire transistors, tunnel transistors, resonant tunneling diodes, and negative capacitance transistors. Our results show that NESS is a robust, fast, and reliable simulation platform which can accurately predict and describe the underlying physics in novel ultra-scaled electronic devices.

## 1. Introduction and State-of-the-Art

Technology computer-aided design (TCAD) tools are an essential part of the research and design in the development of future nano-electronic devices. The use of TCAD tools reduces the research and development costs and time-to-market for the semiconductor industry by taking advantage of the physical insights offered for both already existing devices or novel devices architectures [1]. Many commercial and academic research groups are involved in the development of TCAD toolboxes and computational frameworks. In previous publications [2,3], we have presented in detail the inner workings of the user-friendly TCAD semiconductor device simulation platform called Nano-Electronic Simulation Software (NESS). In this paper, we focus on the illustration of the capabilities of NESS to simulate different device types and architectures. We will demonstrate capabilities of NESS by using the implemented solvers. In general, the NESS’s modular structure allows the use of multiple solvers with different complexity within the same device simulation domain.

**Figure 1:**
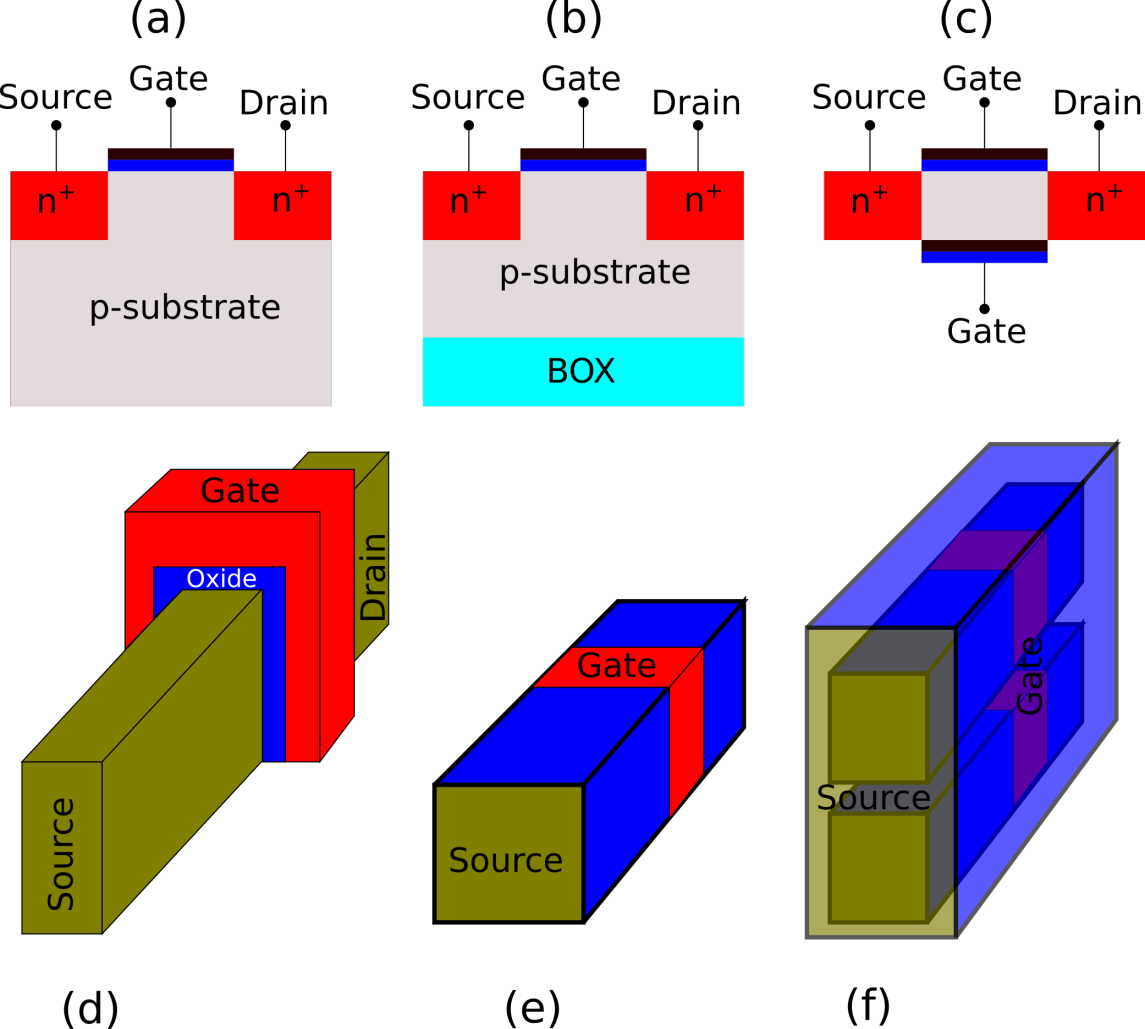
Evolution of CMOS devices. (**a**) planar MOSFET, (**b**) Silicon-On-Insulator (SOI), (**c**) double gate, (**d**) FinFET, (**e**) single Nanowire Transistors (NWT), (**f**) stacked-NWT.

The metal-oxide-semiconductor field-effect transistors (MOSFET) proved to be crucial in the development of smaller transistors and faster electronic circuits and systems. Moore’s law predicts that every two or three years the number of transistors on the chip would double thanks to the MOSFET scaling. However, as the scaling reached the nanoscale regime, the performance of planar MOSFETs starts to significantly deteriorate. To overcome these issues, different techniques were used to allow better control of the channel. In Figure 1, we illustrate the evolution of the MOSFET. For the planar devices, two of the initial solutions were the silicon-on-insulator (SOI) structure and the double gate device. However, in order to keep up with Moore’s law, a novel architecture was adopted by the industry, the so called FinFET. This was the first 3D device to have a non-planar channel. It is predicted that the nanowire transistor could be one of the main future contenders for enabling further scaling. It has the advantage of being fabricated in a stacked configuration and so it can achieve high drive currents in denser silicon chips thus extending Moore’s Law [4]. However, it is crucial to understand the physics and the operation of these novel devices. In this paper, our objective is to show how NESS can help to understand the physics and the behavior of different novel devices, thus aiding and accelerating their research and development.

The devices investigated include: (1) Nanowire Transistors (NWT), which are considered as potential evolution of the current FinFET architecture; (2) Tunneling Field-Effect Transistors (TFET), which have the potential to be used for low power applications thanks to their extremely low sub-threshold swing (SS); (3) Negative Capacitance FETs (NCFET), which improve the performance of transistors at the nano-scale by sub-thermionic switching; and (4) Resonant Tunneling Diodes (RTD), which show a great promise for high-speed applications. In this paper, we demonstrate how all of the above-mentioned devices can be easily simulated and studied using NESS. In the next couple of paragraphs, we provide a brief motivation for the choice of these particular devices.

Since 2011, the introduction and the mass production of non-planar CMOS devices (i.e., FinFET) has demonstrated that the industry is still capable of keeping up with Moore’s Law [5]. Multi-gate device architectures have shown that scaled devices are capable of a better electrostatic control while maintaining a high enough ON-current for improving the circuit performance. However, the further FinFET scaling beyond the 5nm technology node will lead to device characteristics and performance degradation, including severe drain-induce barrier lowering (DIBL), high SS, and variability issues. Therefore, new super-scalable FET architectures are intensively researched. The most likely winner is the NWT, thanks to the all-around gate and the possibility for stacking NWTs [6,7].

One of the disadvantages of CMOS transistors, either planar or non-planar, is the bottom limit of the sub-threshold slope (SS) of approximately 60mV/dec. One of the proposed devices to overcome this limit is the TFET which has demonstrated extremely low SS values theoretical and experimentally [8,9,10,11]. The great advantage of TFETs is that the carrier injection is due to the band-to-band tunneling (BTBT) mechanism, which, in turn, allows the achievement of a very low leakage current [12]. This is ideal for very low-power device applications.

One of the disadvantages of TFETs is their low ON-current. However, NCFETs present an alternative solution to achieve a steep SS while maintaining high ON-current [13]. A simple modification of the already existing CMOS structures [14,15] proves useful as it requires minimal change in the fabrication process. One proposed method is to stack a ferroelectric layer on top of the gate dielectric of the MOSFET [14]. These changes will be beneficial for low-power applications that require high-speed switching [15].

In the past, a great amount of research was invested in RTD that continues into the present [16,17]. One of the great advantages of these devices is negative differential resistance (NDR) characteristics that work in the terahertz (THz) frequency range, which is of interest of applications application such as RF-oscillators, physical unclonable function (PUF) devices and random number generators, including communications [16,17]. Thanks to the huge research effort, RTDs were demonstrated to achieve one of the highest oscillation frequencies at room temperature with respect to other contenders [16,17]. Moreover, it was demonstrated that RTDs have a unique features due to their randomness [18]. These devices can exhibit very different current output, thanks to the intrinsic atomistic nature of the materials and interfaces. It is believed that it can be utilized for realizing physical unclonable digital fingerprints [19,20].

This paper is organized as follows. In Section 2, we provide a brief overview of the NESS structure. In Section 3, we discuss the devices under investigation: Section 3.1 NWTs, Section 3.2 TFETs, Section 3.3 NCFETs, and in Section 3.4 RTDs. Finally, in Section 4, we finish with the concluding remarks.

## 2. Overview of NESS

**Figure 2:**
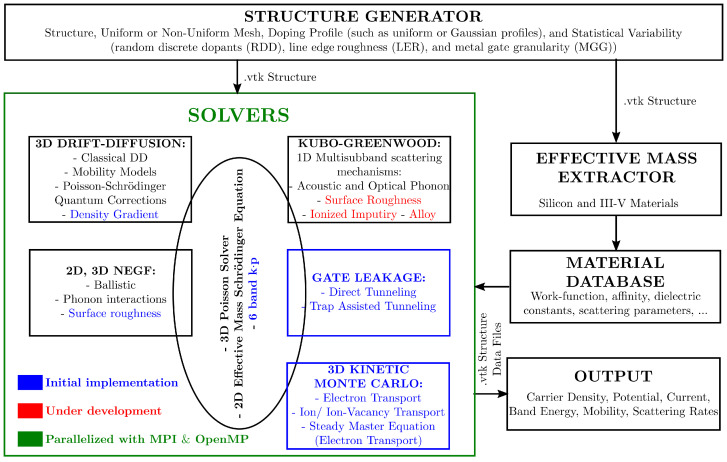
Flowchart of NESS detailing its modular structure.

NESS [2,3,21,22] is a user-friendly TCAD semiconductor device simulator, under development by the Device Modelling group at the University of Glasgow. It has been developed considering three main concepts: (1) NESS enables simulations with increasing complexity and physical content within a unified environment (i.e., it offers the possibility of investigating a particular transistor structure with different complexity of simulations techniques from classical to quantum transport); (2) NESS has been designed to be flexible, easy to use, and extendable thanks to its modular structure; and (3) this modular structure allows for future collaborations and co-developments by industry and academia all over the world. Its modular structure is illustrated in Figure 2, where the five main components of NESS are presented: the structure generator (SG), the effective mass extractor, the material database, the solvers, and the output generator allowing to store the simulation results (i.e., current, electrostatic potential, charge concentration).

Firstly, the SG [23,24] is a flexible module capable of generating and configuring various types of devices (such as NWTs, multi-gate 3D devices architectures, or bulk CMOS transistors) and the corresponding simulation domains. It accepts a text file as input, and the generated device structure can be saved in a binary or ASCII format (vtk files) for easy visualization with freeware software, such as ParaView. The solved simulation domain is saved in vtk format as well (in addition to output text files) and can be similarly visualized and further post-processed in a straight-forward manner. The datasets are defined by the rectilinear grid with a regular topology along the coordinates. It allows users to consider different semiconductor materials (such as Si, Ge, or III-Vs materials), doping configurations (such as uniform or Gaussian profiles), uniform or non-uniform mesh designs, and the main sources of statistical variability in nanoscale devices (random discrete dopants (RDD), line edge roughness (LER), metal gate granularity (MGG)), and trapped charges at the interface or in the dielectric (ITC). Some examples of all these different structures and configurations are described in Section 3.

Secondly, as the effective masses strongly depend on the characteristic dimension and the confinement orientation of the nanostructures, an automated module to extract the effective mass from first principle simulations has been implemented in NESS [25]. It can calculate the correct electron confinement and transport effective masses from atomistic simulations (such as density functional theory (DFT)) or semi-empirical models (such as tight-binding (TB)) of the electronic band structure of NWTs with the technologically relevant cross-sectional area, shape, and transport orientations. The capabilities of this module have been already demonstrated in accurately computing the effective masses of Si [25] and Si${}_{x}$Ge${}_{1-x}$ [23] NWTs considering different dimensions and shapes.

Thirdly, the material database provides the relevant parameters for each material considered in the generated structure, such as the work-function, affinity, dielectric constants, mobility model parameters, or scattering parameters. Furthermore, the effective masses can be provided for each material from DFT and TB methods, or directly from our effective mass extractor. As illustrated in Figure 2, those parameters serve as input for the solvers.

Fourthly, different transport simulation solvers [2,3,21] have been implemented in NESS to simulate the mobility, the charge density, and the current in nano-CMOS devices. All modules have been implemented with a high degree of parallelism making use of MPI and OpenMP libraries. In general, each of them is solved self-consistently with the 3D Poisson and the 2D Schrödinger equations. Currently, there are three main numerical solvers, ranging from classical to quantum transport.

The drift-diffusion (DD) module is indispensable for simulating bulk CMOS transistors and relatively large devices where a more sophisticated approach is neither desired nor practical. The classical solution has been implemented using a finite volume discretization scheme to solve the current continuity equation [26]. Two different improvements have been implemented to enhance this module: (1) different mobility models including the doping dependence of the mobility (Masetti model [27]) and the transverse and longitudinal electric field dependence of the mobility (Yamaguchi model [28] and the Caughey-Thomas [29] model, respectively); and (2) Poisson-Schrödinger quantum corrections [30] to capture the quantum confinement effect at a fraction of the computational cost of a full quantum simulator.

The Kubo–Greenwood (KG) module provides accurate electron mobility at low-field near-equilibrium conditions [31,32,33]. It combines the quantum effects based on the 1D multi-subband scattering rates of the most relevant scattering mechanisms (acoustic and optical phonon, and surface roughness scattering mechanisms) in confined channels [34] and the semi-classical Boltzmann Transport Equation by applying the KG formula within the relaxation time approximation [35]. Two strategies have been implemented to compute the total mobility: (1) it could be calculated as a function of the individual mobilities associated with each scattering mechanism using the Matthiessen rule; or (2) the scattering rates of all mechanisms could be directly added to avoid the Matthiessen rule and, thereby, the total mobility is computed using the KG Equation. The strategy of directly adding the scattering rates to compute the total mobility is of special interest when devices with large cross-sections are simulated due to the high error induced by the Matthiessen rule. On the contrary, for narrower devices, the error induced by the Matthiessen rule is less, with the results computed by multi-subband Monte Carlo and NEGF approaches being comparable.

The coupled mode-space NEGF solver [2,21] allows the quantum treatment of charge transport in order to capture quantum phenomena such as tunneling, coherence, and particle–particle (wave–wave) interactions in mesoscopic and nanoscale devices. It is possible to consider dissipative transport by switching on the acoustic or optical phonon scattering to enable electron-phonon (e–ph) interactions within the self-consistent Born approximation (SCBA) or neglect them to investigate the purely ballistic transport [36,37,38]. Moreover, the NEGF solver implemented in NESS allows to simulate 2D planar structures, such as DGSOI [39], and to calculate the BTBT by using the Flietner model to compute the current in heterostructures with a direct bandgap [40]. A combination of this NEGF module with a full-band quantum transport solver in presence of hole-phonon interactions using a mode-space k·p approach has been also implemented [41].

Finally, different enhanced modules and solvers [22] are currently under development in NESS including: density gradient; extension of the KG module [34] to consider ionized impurity and alloy scattering mechanisms; implementation of surface roughness scattering mechanism in the existing NEGF module [42]; Kinetic MC solver [43,44] for the simulation of memory devices; and a module to compute the gate leakage current.

## 3. Non-Conventional or Future Devices

### 3.1. Nanowire Transistors

As previously discussed in Section 1, different technological architectures have been proposed to overcome the limitations of the FinFET CMOS technology. One of these promising solutions is the use of multiple gates surrounding the channel, which increases the electrostatic confinement and reduces the short-channel effects (SCE) [45], with the potential for replacement of the Triple-Gate FinFET technology at the ultimate scaling limits [46,47,48,49], especially for CMOS scaling beyond the 5 nm node [50]. The advantages of the NWTs include improved charge control in the channel (minimizing SCEs), superior transport properties, and the possibility of using material and strain engineering to improve the device performance.

Focusing on the channel material for future devices, SiGe, III-V, and two-dimensional materials (such as graphene, boron nitride) are attracting attention due to their small transport effective masses (m${}_{trans}^{*}$) [51,52,53,54,55,56]. It is important to mention that materials with smaller m${}_{trans}^{*}$ increases both the ON-state and OFF-state currents (I${}_{ON}$ and I${}_{OFF}$, respectively) due to the source-to-drain tunneling currents. Nevertheless, it is worth noting that a superior material that could replace Si has not yet been identified.

**Figure 3:**
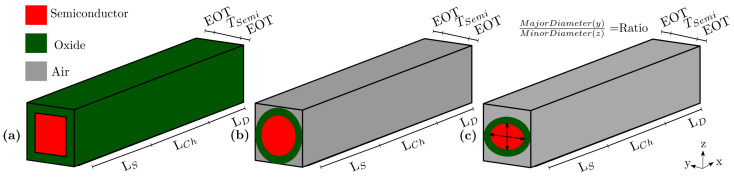
Schematic illustration of nanowire transistors (NWT) for (**a**) square, (**b**) circular, and (**c**) elliptic shapes (with constant diameter) showing the main parameters: source length (L${}_{S}$), channel length (L${}_{Ch}$), drain length (L${}_{D}$), semiconductor thickness (T${}_{Semi}$), and Equivalent Oxide Thickness (EOT). In NESS, the X direction represents the transport direction and the plane YZ represents the 2D confinement plane. For the elliptic NWT, the ratio between the major (y direction) and minor (z direction) diameters is 1.667.

In this section, we have considered SiGe material as a channel to provide an advanced example for NWTs simulation with NESS, because this material is more compatible with the current CMOS technology [57]. In particular, we have simulated n-type Si${}_{x}$Ge${}_{x-1}$ channel NWTs adjusting the material properties of SiGe by changing the mole fraction to have the trade off between the advantages of Si and Ge individually. The chosen structure has L${}_{G}$ = 10 nm and a diameter of 5 nm following the prospect for the year 2024 given by the Institute of Electrical and Electronics Engineers (IEEE) International Roadmap for Devices and Systems (IRDS) report [58]. Moreover, as the charge carriers are confined in these devices in a cross-section perpendicular to the transport direction (YZ plane on Figure 3), NWTs with different shapes have been considered in this example including square, circular, and elliptical (with constant diameter) NWTs in order to show the impact of the shape on the electrostatics [59] and transport. The schematic of the simulated NWTs with different shapes and their main structure parameters are shown in Figure 3.

In order to capture the S/D tunneling transport, the simulations have been performed with the NEGF solver considering only ballistic transport. It is worth mentioning that the benefit in using SiGe for n-type MOSFETs is very small and introduce alloy disorder scattering not present in pure Si. Alloy disorder scattering is not implemented in the current version of NESS and it is not considered in this work. To adopt more realistic conduction bands in this nanoscaled structures, the effective masses have been extracted for each shape from atomistic simulations making use of the effective mass extractor of NESS [23].

**Figure 4:**
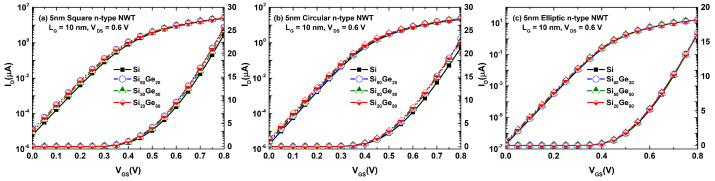
I${}_{DS}$ vs. V${}_{GS}$ characteristics for n-type (**a**) square, (**b**) circular, and (**c**) elliptic 5 nm NWTs assuming different Si${}_{x}$Ge${}_{1-x}$ mole fractions and ballistic NEGF transport simulations with L${}_{G}$ = 10 nm, [100] transport direction, and V${}_{DS}$ = 0.6 V. The effective masses have been computed making use of the effective mass extractor of NESS [25].

The I${}_{DS}$ vs. V${}_{GS}$ characteristics for these devices are shown in Figure 4. The differences in the drain current as a function of the Ge mole fraction in comparison to the pure Si NWT are less pronounced for the elliptic NWT shape (Figure 4c) than for the other NWT shapes (Figure 4a,b). Moreover, it is worth highlighting that the I${}_{DS}$ vs. V${}_{GS}$ characteristics for the elliptic NWTs (Figure 4c) reveal a higher I${}_{ON}$/I${}_{OFF}$ ratio, which is another factor that shows its superior performance in comparison to circular and square NWT shapes [33] (Figure 4a,b, respectively).”

**Figure 5:**
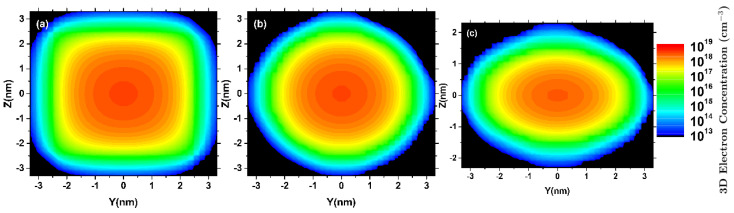
3D profile of electron density in a slice located at the center of the transport direction (X) for n-type (**a**) square, (**b**) circular, and (**c**) elliptic 5 nm × 5 nm NWTs assuming Si${}_{20}$Ge${}_{80}$ fraction and ballistic NEGF transport simulations with L${}_{G}$ = 10 nm, [100] transport orientation, V${}_{DS}$ = 0.6 V, and V${}_{GS}$ = 0.4 V. The effective masses have been computed making use of the effective mass extractor of NESS [25].

Quantum confinement effects modify the electron distribution, determining the charge available for transport [60,61] and, consequently, the electrostatic potential profile [62]. Accordingly, the NWT cross-sectional shape has an important impact on the transistor performance. The impact of the NWT cross-sectional shape on the quantum confinement and, hence, on the electron distribution is shown in Figure 5 where the 3D electron density profile is depicted for a NWT slice located in the center of the transport direction for the device structures of Figure 4 with Si${}_{20}$Ge${}_{80}$ fraction and V${}_{GS}$ = 0.4 V considering (a) square, (b) circular, and (c) elliptic shapes.

**Figure 6:**
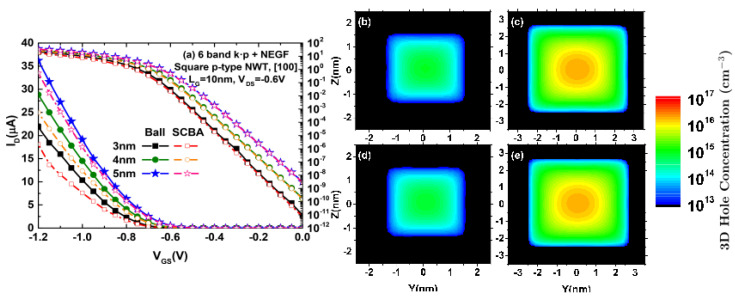
(**a**) I${}_{DS}$ vs. V${}_{GS}$ characteristics for p-type square Si NWTs with 3 nm × 3 nm, 4 nm × 4 nm, and 5 nm × 5 nm cross-sections considering the crystallographic orientation [100] with coherent and dissipative transport. (**b**–**e**) 3D profile of hole density in a slice located at the center of the transport direction (X) for p-type square NWTs with (**b**)/(**d**) 3 nm × 3 nm and (**c**)/(**e**) 5 nm × 5 nm cross-sections assuming (**b**)/(**c**) coherent and (**d**)/(**e**) dissipative transport simulations with L${}_{G}$ = 10 nm, [100] transport orientation, V${}_{DS}$ = −0.6 V, and V${}_{GS}$ = −0.4 V. The results have been simulated making use of the mode-space full-band quantum transport solver included in NESS by combining a six-band k·p Hamiltonian and the existing NEGF module.

The reduction in the confinement dimensions has also a direct impact on the transistor performance. To illustrate this fact, Figure 6 shows (a) the I${}_{DS}$ vs. V${}_{GS}$ characteristics and (b)–(e) the 3D hole distribution of a p-type square Si NWT with cross-section dimension ranging from 3 nm × 3 nm to 5 nm × 5 nm and considering [100] crystallographic orientation with both coherent and dissipative NEGF simulations [2]. The results have been obtained with the mode-space full-band quantum transport solver included in NESS by combining a six-band k·p Hamiltonian and the existing NEGF module. As the geometrical dimensions of an NWT are reduced, the carrier charge tends to be more concentrated in the center of the device, as can be seen when comparing the 3D hole density from (b) 3 nm × 3 nm to (c) 5 nm × 5 nm cross-sectional dimensions in Figure 6.

Apart from the aforementioned impact, the quantum confinement effects, responsible for the modification of the hole distribution in the subbands, directly modify the matrix elements for the coupling with the phonons [41]. This effect can be shown in Figure 6d,e where the dissipative transport have been considered instead of the coherent one (Figure 6b,c). In particular, the phonon scattering mechanism spreads the hole concentration further away from the center of the device due to the phonon interactions.

### 3.2. Tunnel FETs (TFET)

**Figure 7:**
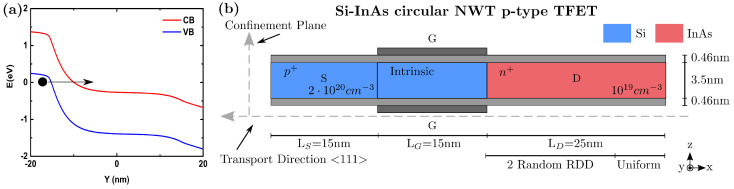
(**a**) Schematic example of the BTBT mechanism in a p-n junction. (**b**) Schematic illustration of the Si–InAs circular p-type TFET along the transport direction considered in this work showing the main parameters: source length (L${}_{S}$ = 15 nm), channel length (L${}_{Ch}$ = 15 nm), drain length (L${}_{D}$ = 45 nm), semiconductor diameter (3.5 nm), Equivalent Oxide Thickness (0.46 nm), and doping. As the tunneling is mainly happening at the intrinsic Si–InAs interface, random dopants distribution (RDD) is only considered in the InAs region. A uniform doped region of 20 nm is considered near the drain contact to ensure convergence.

Over the last decade, the interest in BTBT devices has dramatically increased due to their sharp switching characteristics. The carrier transport in these types of devices is governed by quantum mechanical tunneling through a barrier between energy bands (BTBT), contrary to MOSFETs, where thermionic emission dominates the transport. Figure 7a shows a schematic example of the BTBT mechanism in a pn junction: the high electric fields (>10${}^{6}$ V/cm) across a reverse-biased pn junction causes significant current to flow through the energy barrier due to tunneling of electrons (holes) from the valence (conduction) band of the p (n) region to empty states in the conduction (valence) band of the n(p) region, respectively.

The most popular BTBT devices are the TFETs which, in theory, could achieve sub-thermal SS, i.e., SS < 60 mV/decade. The best MOSFET implementations cannot bring SS below 60 mV/decade, which leads to difficulties [63,64] with the limitation in the V${}_{DD}$ reduction. The main drawback of the TFETs is the lower I${}_{ON}$ in comparison to conventional MOSFETs. However, these devices present a higher I${}_{ON}$/I${}_{OFF}$ ratio, which makes them potential candidates for low-power electronic applications [10,11,65].

At the nanometer scale, and in presence of tunneling, quantum transport simulations are required to accurately model the TFET operation and predict its performance. Few analytical models exist in the literature to compute the BTBT accounting for quantum effects, which are commonly implemented in semi-classical tools based on the Wentzel, Kramers, and Brillouin (WKB) approximation [66,67,68]. In NESS, it is possible to compute the direct BTBT in nanodevices making use of the coupled mode-space NEGF scheme within the effective mass approximation (EMA) and the Flietner model of the imaginary dispersion [40].

Hetero-TFETs made of III-V semiconductors on Si were proposed to increase the I${}_{ON}$ due to the reduction in the BTBT barrier [10,11,69,70]. Accordingly, a Si–InAs circular NWT p-type TFET has been simulated in this work. In this particular structure, the BTBT occurs between the Si–InAs interface and it is mainly direct without phonon-assisted tunneling [66]. Figure 7b shows the schematic of the simulated TFET with the following parameters: L${}_{G}$ = 15 nm, NWT diameter 3.5 nm, [111] transport orientation, and V${}_{DS}$ = −1.0 V. The Si–InAs interface has been considered ideal and abrupt. It has been simulated making use of the NEGF module and the Flietner model only considering ballistic transport. Although the inclusion of phonon scattering could lead to more accurate prediction in most cases, its general impact can be negligible in this particular TFET configuration as it only slightly increases I${}_{OFF}$ [71].

Since the BTBT strongly depends on the energy bands, the potential variations locally modifying the tunneling probability due to the RDDs limit the hetero-TFET performance. It has already been demonstrated that RDDs have more impact on TFETs than the rest of the sources of variability [40], especially for I${}_{ON}$. Accordingly, a second study has been performed here considering several configurations of RDDs in the above-mentioned TFETs. As the tunneling is mainly happening at the Si–InAs interface, the RDDs are only considered in the drain region (InAs part of the device). As depicted in Figure 7b, the RDD region is 20 nm long, and a uniformly doped region of 25 nm length is considered near the drain contact to ensure numerical stability (making the total drain region 45 nm). The number of dopants in each of the TFETs is randomly chosen from a Poisson distribution, with the mean determined by the doping concentration multiplied by the volume of the RDD region. The dopants are then randomly placed using a probability rejection technique.

**Figure 8:**
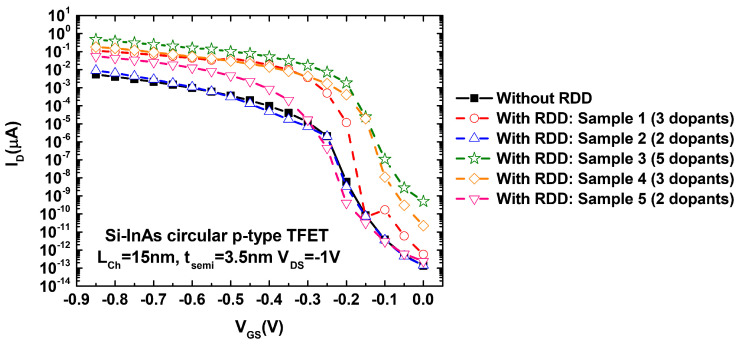
I${}_{DS}$ vs. V${}_{GS}$ characteristics for the Si–InAs circular p-type TFET (schematic shown in Figure 7b) considering a simulation with a uniform doping profile (i.e., without random discrete dopants (RDD)) and five simulations with random distributed dopants. These 5 configurations consider random location of the doping along the 25 nm drain region (Figure 7b) and random number of dopants: Sample 1–5 has 3/2/5/3/2 dopants, respectively.

Figure 8 shows the I${}_{DS}$ vs. V${}_{GS}$ characteristics of the simulated Si–InAs TFETs without RDD (uniform doping profile) and with five random configurations of RDDs. These five random samples have 3, 2, 5, 3, and 2 dopants, respectively. We can easily observe the main advantage of the TFETs by the 10${}^{10}$ I${}_{ON}$/I${}_{OFF}$ ratio of the device without RDD. Moreover, it is worth mentioning that the variability in the I${}_{OFF}$ has approximately three orders of magnitude difference, whereas the I${}_{ON}$ has around two orders of magnitude difference. The figure also shows the increase in the current when the number of random discrete dopants is higher.

**Figure 9:**
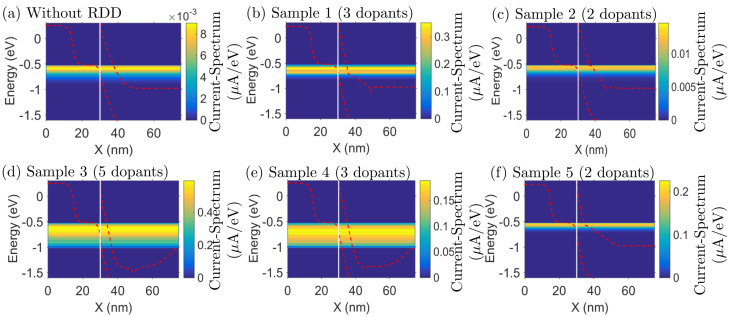
Simulated ON-state (V${}_{GS}$ = −0.65 V) current-spectra of the Si–InAs circular p-type TFET (schematic shown in Figure 7b) considering (**a**) a simulation with a uniform doping profile (i.e., without random discrete dopants (RDD)) and (**b**–**f**) five simulations with random distributed dopants. These 5 configurations consider random location of the doping along the 25 nm drain region (Figure 7b) and random number of dopants: (**b**–**f**) Sample 1–5 has 3/2/5/3/2 dopants, respectively. The red dashed-lines denote the highest valence and the lowest conduction subbands. The vertical white solid-line indicates the Si–InAs interface.

The impact of this variability source can be easily seen in the energy band bending due to the RDD when comparing the current-spectra and conduction and valence bands for the device without dopants (Figure 9a) and with the five different dopant configurations (Figure 9b–f). The current-spectra shown in Figure 9 represents the ON state (V${}_{GS}$ = −0.65 V) showing that the ON-state in a TFET is still controlled by the BTBT barrier length. In the presence of RDD, the barrier width and height becomes localized and depends on the number of dopants and their position.

### 3.3. Negative Capacitance FETs (NCFET)

Negative capacitance (NC) based field-effect transistors are constructed by introducing a source of negative capacitance in the gate stack of a conventional transistor. They have attracted a lot of attention during the last decade as they can overcome the room temperature Boltzmann limit of 60 mV/decade SS in MOSFETs [13]. NCFETs are being viewed as a means to enable significant supply voltage scaling, thus lowering the off-state leakage while enhancing the drive current at the same time due to an effective voltage amplification [72]. NCFETs have been realized in various architectures, including FinFET [73] and nanowire FET [74]. In addition to the prospects of improved nominal device performance [75], NCFETs have been shown to be more immune to statistical variability compared to their conventional counterparts [76,77,78,79]. NCFETs also have shown promising circuit applications [80,81].

Although several approaches for modeling of NCFETs have emerged [82], there is no definitive clarity yet on the physical phenomenon behind the negative capacitance in NCFETs. A popular way to model NCFETs is using the phenomenological Landau–Devonshire (L-D) theory [83] (the time-dependent version of which is the Landau–Khalatnikov theory [84]) for the ferroelectrics. It relates the polarization (*P*) and electric field (*E*) across it in terms of the Landau coefficients that characterize the ferroelectric material and thickness of the ferroelectric ($t_{fe}$). According to the L-D theory, the P−E curve can traverse an *S* shaped trajectory having a negative slope signifying the negative capacitance. Efforts of experimental observation of negative capacitance have succeeded recently with recording of *S*-shaped P−E curve [85,86].

In NESS, we have developed an NCFET module consisting of the steady-state negative capacitance model based on the analytical L-D theory for NCFETs with the Metal-Ferroelectric-Metal-Insulator-Semiconductor (MFMIS) architecture [87]. A simplifying but valid assumption that we have used is that the ferroelectric polarization is equal to the gate charge density, i.e., $P\approx Q_{G}$. In this structure, due to the presence of the internal metal layer, the NCFET can be viewed as a combination of a ferroelectric capacitor connected in series to the metal gate of a conventional transistor (the ‘internal’ or reference MOSFET). As a result, in our analytical or ‘lumped’ approach, the NCFET can be modeled by modeling the internal MOSFET and the ferroelectric capacitor separately and then using Kirchhoff’s voltage law to relate the potential at the internal metal gate to the externally applied bias. This enables us, in the spirit of the modular nature of NESS, to use any of the existing transport solvers in conjunction with the NCFET module. We first obtain $Q_{G}-V_{G}$ and $I_{D}-V_{G}$ characteristics of the internal MOSFET. The NCFET module then calculates the voltage drop across the ferroelectric, $V_{fe}$ using the L-D model, and generates characteristics of the NCFET. Note that here the ferroelectric has been assumed to be monodomain, and the gate leakage [88] has been ignored. This Landau model based approach has been shown to be able to match experimental results including circuit performance in negative capacitance based FinFETs [89,90].

**Figure 10:**
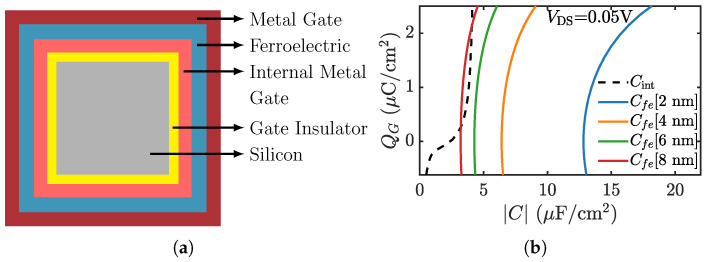
(**a**) Cross-sectional schematic of the MFMIS nanowire NCFET. Device dimensions: Gate length = 10 nm, Channel width = 3 nm, oxide thickness = 1 nm, Source/Drain length = 10 nm. The ferroelectric thickness has been varied up to 8 nm. The parameters used for the ferroelectric are: Coercive field, $E_{c}$ = 1.2 MV/cm and remanent polarization, $P_{r}$ = 8 μC/cm${}^{2}$ which fall in the range of metal doped HfO${}_{2}$-the material of choice for CMOS compatible NCFET technology [94]. (**b**) The capacitance of the internal MOSFET ($C_{\mathrm{int}}$), and magnitude of the ferroelectric capacitance ($C_{fe}$) for different ferroelectric thicknesses, plotted with respect to gate charge density.

**Figure 11:**
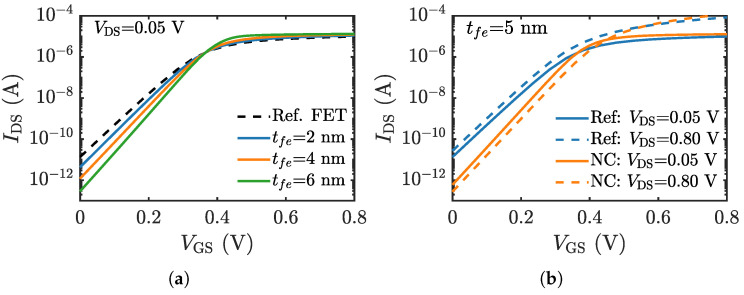
(**a**) $I_{\mathrm{DS}}-V_{\mathrm{GS}}$ characteristics of the reference MOSFET and NW NCFET for $t_{fe}$ = 2, 4, 6 nm at $V_{\mathrm{DS}}$ = 0.05 V. (**b**) The conventional MOSFET shows positive DIBL while NCFETs can display negative DIBL, i.e., increase in the threshold voltage at high drain bias.

As a demonstration of the NCFET module, we have simulated a negative capacitance nanowire FET. Figure 10a shows the cross-sectional schematic of the device. The device dimensions and ferroelectric properties are mentioned in the figure caption. The drift-diffusion module has been used to simulate the carrier transport in the internal NWFET. The key to achieving superior performance in the NCFET is the voltage gain at the internal node, which depends on the capacitance matching between the internal MOSFET capacitance ($C_{\mathrm{int}}$) and the ferroelectric capacitance ($C_{fe}$). Figure 10b shows the two sets of capacitances. The capacitance matching is better when the difference between the internal capacitance and the magnitude of the ferroelectric capacitance is smaller, which occurs when the thickness of the ferroelectric layer is increased, as can be seen in Figure 10b. Note that at very large $t_{fe}$, e.g., 8 nm in Figure 10b, the negative capacitance of the ferroelectric can become smaller than the internal capacitance ($|C_{fe}|$ < $C_{\mathrm{int}}$) which is a region of instability and manifests as hysteretic jumps in the characteristics. The transfer characteristics obtained with varying ferroelectric thicknesses, including the reference NWFET characteristics are shown in Figure 11a. These NW NCFETs show better subthreshold swing, lower off-state leakage, and higher drive currents—typical for short channel multigate NCFETs [75], the characteristics improving with increasing $t_{fe}$. Additionally, in the case of NCFET, under suitable conditions, the source and channel barrier rises when increasing the drain voltage, and, hence, it can have negative DIBL as illustrated in Figure 11b. Note that negative DIBL has been earlier demonstrated theoretically e.g., [75,91], as well as experimentally e.g., [92,93].

A 1D L-K model for the ferroelectric version that can be used for simulating the Metal-Ferroelectric-Insulator-Semiconductor (MFIS) NCFET architecture is under development and we plan to implement the 3D Landau–Ginzburg formalism, and also explore alternative approaches in the near future.

### 3.4. Resonant Tunneling Diodes (RTD)

The RTDs are typical one-dimensional quantum structures that have attracted increasing attention due to their advantages associated with the nanometer-scale semiconductor physics. A basic double-barrier RTD configuration consists of a thin quantum well (QW) made from a small-bandgap semiconductor (GaAs or GaN) sandwiched between two large-bandgap (rectangular) semiconducting tunneling barriers (AlGaAs or AlGaN) [95,96], which are accompanied by two highly doped small-bandgap semiconducting contacts acting as the source and the drain. The RTDs operation is governed by the quantum mechanical tunneling of the incident charge carriers through the potential barrier into the quantized sub-band states within the QW, which leads to resonances in the transmission spectrum. Moreover, by increasing the bias voltage beyond the resonance peak, the tunneling current drops to the valley point, and the RTDs, therefore, will exhibit a non-monotonic behavior in the current-voltage (I–V) characteristics referred to as negative differential resistance (NDR) [97,98].

In electronic circuits, the NDR offers new possibilities in both digital and analog designs by reducing power consumption. Owing to the ultra-high frequency capabilities and relatively simple structure, the RTDs are one of the most promising devices to date for wireless and optical THz generation and detection, hence addressing the feasibility of a wide range of concepts and devices involving THz communications [16,99,100,101,102,103,104,105,106,107] and 5G/6G technology. Recently, the RTDs have been proposed as random number generators [108] and the main building blocks for the quantum-confinement (QC) based physically unclonable functions (QC-PUF) [18]. The resonance voltage in the I–V characteristics depends on the QC within the nanostructure. On the other hand, the QC itself depends on the atomic-scale structure of the device and the corresponding random variability sources which consequently makes the locations of the resonance peaks unique, reliable, random, and extremely difficult to clone [109].

**Figure 12:**
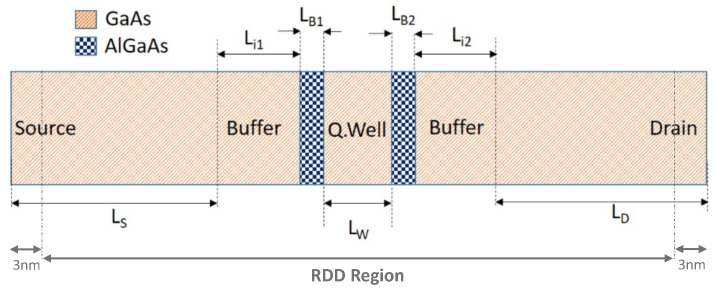
Schematic illustration of the investigated GaAs-Al${}_{x}$Ga${}_{1-x}$As nanowire RTD device with a square cross-section (10 nm × 10 nm) and a total length of 55 nm.

In this section, we present a purely ballistic simulation of the typical electronic and transport quantities of a double barrier III-V RTD device implemented in a nanowire structure shown in Figure 12. All simulations are performed self-consistently utilizing the recursive Green’s function algorithm [110,111] implemented in the NEGF module of NESS.

At the central region of the device, we consider two 3 nm thick regions ($L_{B1,B2}$) made of Al${}_{0.3}$Ga${}_{0.7}$As as the tunneling barriers, enclosing a 5 nm thick GaAs section ($L_{w}$) which serves as the QW. These are separated from the source and drain ($L_{S,D}$ = 19 nm) by buffer regions ($L_{i1,i2}$) having a thickness of 3 nm each. The source and drain regions are n-type doped with a high doping concentration of 2 × 10${}^{18}$ cm${}^{-3}$, whereas the rest of the device is taken as intrinsic.

**Figure 13:**
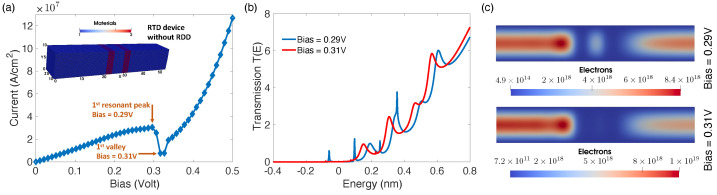
(**a**) The I–V characteristic of the RTD device, where the inset shows its 3D simulation domain in NESS. (**b**) The comparison of the transmission spectra for the first resonant peak (V${}_{R}$ = 0.29 V) and the first valley (V${}_{R}$ = 0.31 V). (**c**) The charge distribution in the XY cut-plane of the device for the first resonant-peak and the first valley.

As depicted in Figure 13a, the current through the nanowire RTD rises steadily to reach the first resonance peak at a bias of 0.29 V, followed by a sharp drop in current down to the first valley at the post-resonance bias of 0.31 V. By applying bias, three distinct regions-first positive-, negative-, and second positive-resistance regions-are being formed within the I–V characteristic. Firstly, for low biases, as bias increases the first confined state (resonant state) between potential barriers gets closer to the source Fermi level, and, therefore, the current it carries increases. Secondly, by further increasing the bias, the first confined state becomes lower in energy and gradually goes into the energy range of the bandgap, and, therefore, the current it carries drops abruptly. The second confined state is still too high in energy to conduct any significant current. Lastly, upon further increasing the bias, as the second confined state becomes closer to the source Fermi level, it carries more current, therefore, current increases similarly to the first positive resistance region. In order to explain this exotic I–V profile referred to as the NDR region, we have shown the transmission spectra T(E) in a 2D cut along the transport direction in Figure 13b. For the resonance bias V${}_{R}$ = 0.29 V, there is a peak at −0.059 eV in the T(E) that vanishes for the post resonance bias of 0.31 V and causes the above-mentioned NDR region.

Moreover, as depicted in Figure 13c, the charge distribution in the device also shows a significant discharging of the QW in the post-resonance condition, which is consistent with the T(E) profiles.

**Figure 14:**
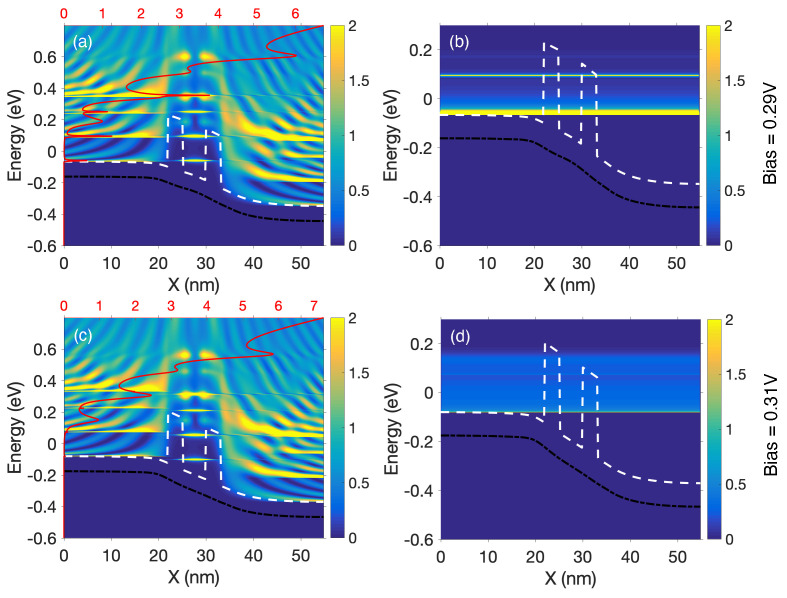
(**a**–**c**) The combined plots of the LDOS, potential, first sub-band, and transmission spectra T(E), and (**b**–**d**) the energy-resolved current spectra of the device at the first resonance peak (V${}_{R}$ = 0.29 V) and the first valley (V${}_{R}$ = 0.31 V). The red lines display the transmission, the white dashed lines indicate the energy sub-band structure, and the black solid lines correspond to the average potential. Here, X is the transport direction, and the red tick marks on the horizontal axes correspond to the magnitude of the T(E).

The alignment of the discrete states between the source side, the well, and the drain side at the bias of 0.29 V is quite clear from the local density of states (LDOS) shown in Figure 14a, thus concurring with the I–V characteristics and the sharp transmission peaks depicted in Figure 13a,b. The T(E), which is shown superimposed on the LDOS with the red solid line, indicates that these peaks are specifically located at the energies at which there is an alignment of the states at −0.059, 0.096, and 0.355 eV for the bias condition V${}_{R}$ = 0.29 V. This alignment is very sensitive to the applied drain bias and vanishes when it is increased to the post-resonance voltage (0.31 V), see Figure 14c. The corresponding current spectra at Figure 14b–d, also reveals the resonant tunneling phenomena by the two very prominent lines at two such aligned energy states, and more discrete tunneling current contribution. The tunneling across the potential barriers is also very prominent in these simulation results. When the device is driven beyond the resonance condition, it is clearly visible that the current spectra becomes smaller in magnitude and rather smeared in nature. Although tunneling across the barrier still exists, there is an absence of the sharp lines in the transmission spectra and the current spectra.

**Figure 15:**
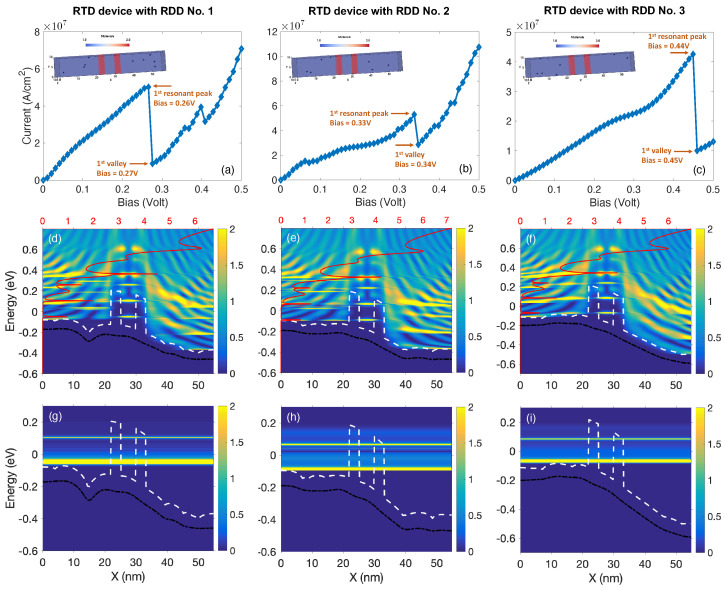
(**a**–**c**) The I–V characteristics of three random RTD devices in the presence of RDDs, where the insets depict the exact positions of the dopants within the RDD region of the structure (see Figure 12) as the spherical balls. The middle row, (**d**–**f**), shows the combined LDOS and T(E) of these RDD devices calculated for the bias at which the first resonant peaks occur. Accordingly, the bottom row (**g**–**i**) presents the corresponding energy-resolved current spectra of the aforementioned devices.

Following on from these results, we have investigated the impact of RDDs on the resonance voltage in nanowire RTDS. For this purpose, we have simulated a sample of three devices with randomly distributed dopants in the RDD region indicated within the baseline structure in Figure 12. The I–V characteristics of the investigated RTD devices in presence of RDDs are shown in Figure 15a–c. From the insets, we see that devices No. 2 and No. 3 contain 7 dopants each with different spatial distribution, whereas device No. 1 contains 9 dopants. Comparing their I-V curves with the I–V characteristic of the baseline device in Figure 13a, we clearly see that they differ from each other in terms of (1) position of the resonant-peak, (2) position of the valley, and (3) the magnitude of the current, therefore making them unique and distinguishable from one another in terms of the signal processing. In order to confirm and compare the effect of the RDDs on the observable characteristics of the RTD results presented in Figure 13, in Figure 15 we have plotted the energy sub-band structure, the transmission spectra, the LDOS, and the current spectra of the three aforementioned devices with RDDs under study. We observe significant differences owing to the number and also the position of the random dopants.

In terms of the sub-band structures (white dashed lines) and the average potential (black solid lines), the changes due to the dopants are very much visible. We also see that the T(E), indicated as red solid lines, spike at different energy levels, and also the current conduction through the channels have different weight for each case.

Taking advantage of the unique capabilities of NESS, we have shown that the quantum nature of the resonance condition can be significantly altered due to the RDDs variability in the III-V nanowire RTD structures. There is a direct link between the number and positions of the RDDs, and the position of the resonance peak. Our results demonstrate that NESS can be used to predict and tailor RTD behavior for various applications.

## 4. Conclusions

In this paper, we have illustrated the capabilities of NESS, a flexible nano-electronic device simulator, to simulate advanced nanoscale semiconductor devices. Based on the discussion and the results in this paper we have shown that NESS is capable of simulating various novel device architectures such as nano-wires, resonant tunneling diodes, tunneling field-effect transistors, and negative capacitance transistors. All structures are created by using the NESS structure generator that enables the generation of semiconducting devices with different architectures, sizes, and shapes. Our structure generator also can introduce the relevant sources of statistical variability, such as random dopants in the corresponding solution domains. In this paper, most of the numerical simulations are executed by using our quantum transport module based on non-equilibrium Green’s function (NEGF) formalism. Here, we have proved that NESS is capable of capturing complex quantum mechanical effects such as quantum confinement, tunneling through the barrier, and band-to-band tunneling. Additionally, NESS is capable of calculating the eigenvalues and eigenvectors in devices with quantum wells such as resonant tunneling diodes. Hence, NESS is very well suited to simulate state-of-the-art nano-electronic devices with various structures where quantum mechanical effects are playing a major role in transport and device performance.

## Data Availability

Data available on request due to restrictions eg privacy or ethical.
